# Glucose Metabolism, Renal Indices, and C‐Reactive Protein Across Two Cesarean Deliveries in the Same Women: A Retrospective Paired Cohort Study

**DOI:** 10.1155/jdr/2449846

**Published:** 2026-11-06

**Authors:** Yu Zhang, Yan Li, Chunling Ma, Jie Chen, Qun Gao, Chao Li, Lin Xu, Rui Chen

**Affiliations:** ^1^ Department of Obstetrics and Gynaecology The University of Qingdao Qingdao China unicamp.br; ^2^ Department of Obstetrics The Affiliated Hospital of Qingdao University Qingdao Shandong China qdu.edu.cn; ^3^ Department of Reproductive Medicine The Affiliated Hospital of Qingdao University Qingdao Shandong China qdu.edu.cn

**Keywords:** C-reactive protein (CRP), cesarean delivery, gestational physiology, glucose metabolism, intraindividual comparison, lipid profile, longitudinal analysis, oral glucose tolerance test (OGTT), pregnancy biomarkers, renal function

## Abstract

**Background:**

Pregnancy is accompanied by changes in glucose metabolism, renal function, and inflammatory activity. However, how these markers differ between successive pregnancies in the same woman after cesarean delivery remains unclear.

**Objective:**

The aim of this study is to characterize intraindividual changes in glucose metabolism, renal function, and inflammatory markers between two cesarean‐delivered pregnancies in the same cohort of women.

**Methods:**

We conducted a retrospective review of 108 women who underwent two singleton cesarean deliveries at the same hospital between 2012 and 2022. Glucose was measured for each pregnancy during a 75 g oral glucose tolerance test at 24–28 weeks of gestation, and C‐reactive protein (CRP) was assessed 24–48 h after delivery. Third‐trimester measurements included serum urea, creatinine, uric acid, lactate dehydrogenase, and the blood urea nitrogen‐to‐creatinine ratio. Paired *t*‐tests or Wilcoxon signed‐rank tests were used as appropriate.

**Results:**

Relative to the first pregnancy, the second pregnancy was characterized by higher 2‐h plasma glucose after a 75‐g glucose load (median 5.99 mmol/L vs. 6.03 mmol/L, *p* = 0.011), lower maternal weight (*p* = 0.008), BMI (*p* = 0.028) and birth weight (*p* = 0.034), an increased BUN/Scr (*p* < 0.001), and lower serum creatinine (median 67.7 vs. 65.1 *μ*mol/L, *p* < 0.001). No significant differences were observed for CRP, fasting glucose, or other renal indicators.

**Conclusions:**

Compared with the first pregnancy, the second pregnancy was associated with higher 2‐h plasma glucose after a 75‐g glucose load, whereas postoperative CRP levels were not statistically significant between the first and second cesarean deliveries. These findings suggest that glucose handling and renal indices may change between pregnancies and warrant further investigation in future studies.

## 1. Introduction

Pregnancy is accompanied by changes in glucose metabolism, renal function, and inflammatory activity as the maternal body adapts to support fetal growth [1]. Disturbances in these processes may increase the risk of adverse maternal outcomes. These changes are necessary to meet the metabolic demands of the growing fetus but may also predispose mothers to health risks when dysregulated. Gestational diabetes mellitus (GDM), defined as glucose intolerance first recognized during pregnancy, affects approximately 7%–14% of pregnancies worldwide and is associated with an increased risk of preeclampsia, macrosomia, operative delivery, and long‐term development of Type 2 diabetes mellitus (T2DM) in mothers and offspring [2–5]. Although most research focuses on single pregnancies, less is known about how these metabolic and biochemical parameters evolve between consecutive pregnancies in the same individual after a cesarean section (CS).

CS is one of the most common surgical procedures worldwide, with rising rates in many regions [6–8]. Beyond its obstetric implications, CS induces a standardized systemic inflammatory and metabolic stress response, characterized by increased circulating C‐reactive protein (CRP) and other acute‐phase reactants in the immediate postoperative period [9], leading to postpartum infection, degeneration of the arcus tendineus fascia pelvis and other complications [10]. Comparing successive pregnancies in the same woman allows assessment of changes in inflammatory and metabolic markers while reducing confounding from stable individual characteristics, such as genetic background and baseline metabolic tendency. However, changes in lifestyle and other time‐dependent factors may still affect these measures.

Emerging evidence suggests that specific oral glucose tolerance test (OGTT) parameters may be sensitive markers of *β*‐cell dysfunction and hepatic insulin resistance during pregnancy, even in women without GDM [11–13]. Similarly, renal function undergoes a significant hemodynamic adaptation in pregnancy [14], with increased glomerular filtration rate (GFR) leading to reduced serum creatinine, and the blood urea nitrogen‐to‐serum creatinine ratio (BUN/Scr) may reflect subtle shifts in protein metabolism, renal perfusion, or tubular handling. [15]. However, little is known about whether these parameters shift in a consistent pattern across pregnancies in the same woman with a prior CS, and how such changes might relate to subsequent metabolic or cardiovascular risks.

In this retrospective paired cohort study, we compared glucose tolerance, postoperative CRP, and renal indices between the first and second cesarean deliveries in the same women over a 10‐year period. We aimed to describe changes in these markers between pregnancies and assess their potential relevance to clinical monitoring in subsequent pregnancies.

## 2. Methods

### 2.1. Study Design

We conducted a retrospective, intraindividual, longitudinal cohort study to evaluate changes in glucose metabolism, renal function and inflammatory markers between two consecutive cesarean‐delivered pregnancies in the same women. The study was performed at a tertiary medical center in China and enrolled participants with deliveries from 2012 to 2022. All procedures complied with the Declaration of Helsinki, and approval was obtained from the institutional review board (QYFY‐WZLL‐29236). Given the use of deidentified data, the requirement for written informed consent was waived.

The intrasubject design minimized the influence of unmeasured confounders, such as genetic predisposition, early life exposure and baseline metabolic status. These are limitations that are often encountered in cross‐sectional or case–control studies.

### 2.2. Participants

Eligible women had two singleton cesarean deliveries at the same hospital, with an interpregnancy interval of 1–10 years. Women with pregestational diabetes, chronic kidney disease, autoimmune disease, major cardiovascular disease, or GDM in the first pregnancy were not eligible. Multifetal pregnancies were also excluded, as were cases involving suspected or confirmed infection within 72 h before CRP measurement, postoperative complications, blood transfusion during or after surgery, or missing core laboratory measurements (CRP or 75‐g OGTT results data). Other laboratory variables were not part of the eligibility criteria, and missing data for these variables were allowed. Analyses using these variables included participants with available data, and the exact number of participants included in each analysis was reported in the figure legends (Figure 1). The diagnostic criteria for postoperative infection were defined as follows: (1) axillary temperature higher than 38.0°C on two measurements taken 4 h apart, accompanied by chills; (2) abnormal manifestations of the abdominal incision, including redness, swelling, tenderness, exudation and local hyperthermia; (3) persistent lower abdominal pain, uterine tenderness or deep pelvic tenderness; (4) purulent lochia with a fetid odor; (5) clinical signs of respiratory tract infection, urinary tract infection, mastitis or lower extremity phlebitis; (6) marked elevation in white blood cell count (>15 × 10^9^/L or persistent elevation on serial daily measurements), neutrophil percentage (> 80% or persistent elevation on serial daily measurements) or procalcitonin levels (> 0.50 ng/mL or persistent elevation on serial daily measurements). Importantly, the diagnosis of postpartum infection was made through comprehensive assessment integrating clinical manifestations and laboratory findings. Finally, 108 patients without GDM in the first pregnancy were enrolled, among whom 97 had no GDM in the second pregnancy, whereas 11 were diagnosed with GDM in the second pregnancy. Maternal age, predelivery maternal weight/body mass index (BMI), gestational age at delivery, and neonatal birth weight were obtained from medical records. To elucidate the impact of a previous cesarean delivery on glucose metabolism and renal function parameters in a subsequent pregnancy, this study included only scheduled cesarean delivery cases.

**Figure 1:**
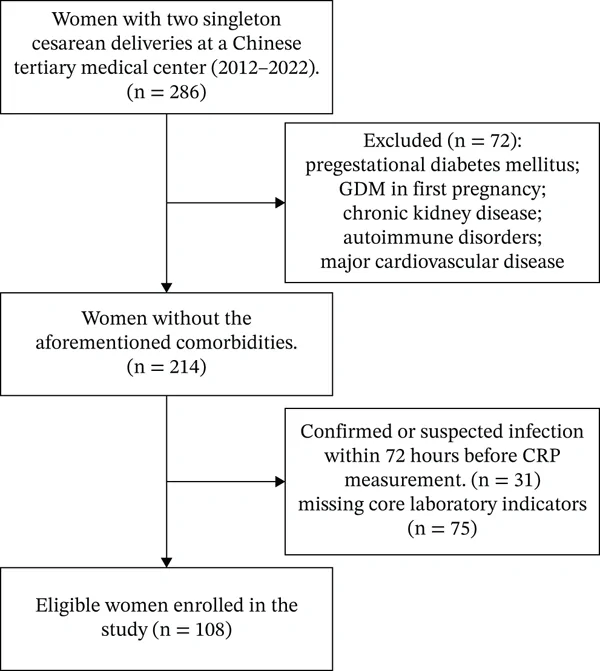
A flow‐chart of selection and exclusions. Women were eligible if they had undergone two singleton cesarean deliveries at a tertiary medical center in China and included deliveries between 2012 and 2022. A total of 108 women were included in the study after meeting the eligibility criteria and being screened for exclusion.

### 2.3. Data Collection

We used the hospital′s computer system to find the results of tests done in each woman′s pregnancy. To assess maternal glucose utilization, we performed a 75‐g OGTT after an overnight fast between 24 and 28 weeks of gestation. [16]. We measured maternal plasma glucose levels at 0, 1, and 2 h during the 75‐g OGTT. Serum CRP was measured 24–48 h postcesarean to capture the peak postoperative inflammatory response while avoiding labor‐related effects. Renal function was evaluated using serum creatinine, urea, and uric acid measurements, from which the BUN/Scr was calculated; lactate dehydrogenase (LDH) was included as an indicator of metabolic and tissue stress. Data on renal function, age, maternal weight, and BMI were collected the day before cesarean delivery. All tests were done in the hospital′s main laboratory using standard methods and equipment. The specific testing methods for each indicator are as follows: glucose: glucose oxidase method; CRP: nephelometric method (scatter turbidimetry); creatinine: sarcosine oxidase method; blood urea nitrogen (BUN): urease method; LDH: lactate–pyruvate method; uric acid: uricase–peroxidase method; urea: UV–glutamate dehydrogenase method. The company has internal and external quality control procedures that meet national and international standards.

### 2.4. Statistical Analysis

The data was analyzed using SPSS Version 27. The Shapiro–Wilk test was used to check if the data followed a normal distribution. Variables that were normally distributed were expressed as the mean ± the standard deviation (SD) of the variable, and compared using paired‐sample *t*‐tests. Variables that were not normally distributed were presented as medians with interquartile ranges (IQRs) and compared using a special test called the Wilcoxon signed‐rank test. We used linear mixed‐effects models to adjust for maternal age, interpregnancy interval, BMI change, and gestational age at laboratory assessment. It was employed with a compound symmetry covariance matrix for repeated measurements. Outcome variables comprised CRP, OGTT and renal function indices. The maternal age, interpregnancy interval, BMI change, and gestational age at blood sampling were included as covariates. Statistical significance of fixed effects was evaluated using Type III tests. A two‐tailed *p* value of less than 0.05 was considered statistically significant. In line with recommendations for reporting observational studies, missing values were reported without imputation.

## 3. Results

### 3.1. Result 1: Inflammatory Markers and Glucose Metabolism Changes Between Pregnancies

Among women with cesarean in their first pregnancy, postoperative serum C‐reactive protein (sCRP) levels showed no statistically significant difference between the first and second cesarean deliveries (median 62.58 mg/L vs. 65.44 mg/L, *p* = 0.126, Figure 2A, Table S1), indicating a stable acute‐phase inflammatory response despite repeated surgical stimulation. In addition, no meaningful changes were observed in fasting glucose (mean 4.32 mmol/L vs. 4.36 mmol/L, *p* = 0.286, Figure 2B). In contrast, the 1‐h plasma glucose and 2‐h glucose concentrations after a 75‐g glucose load at 24–28 gestational weeks were significantly higher in the second pregnancy than in the first (1‐h glucose: mean 6.81 mmol/L (first pregnancy) vs. 7.43 mmol/L (second pregnancy), *p* < 0.001, Table S2; 2‐h glucose: median 5.99 mmol/L (first pregnancy) vs. 6.03 mmol/L (second pregnancy), *p* = 0.011, Figure 2C,D). Correspondingly, these findings were replicated in the cohort without GDM in both their first and second pregnancies (Table S3). Furthermore, we used linear mixed models, adjusting for baseline maternal age, increase in age between pregnancies, change in BMI, and gestational age at predelivery laboratory testing, which occurred 1 day prior to delivery. These analyses confirmed a statistically significant difference in 2‐h plasma glucose after a 75‐g glucose load between pregnancies (*p* = 0.033, Table S4).

**Figure 2:**
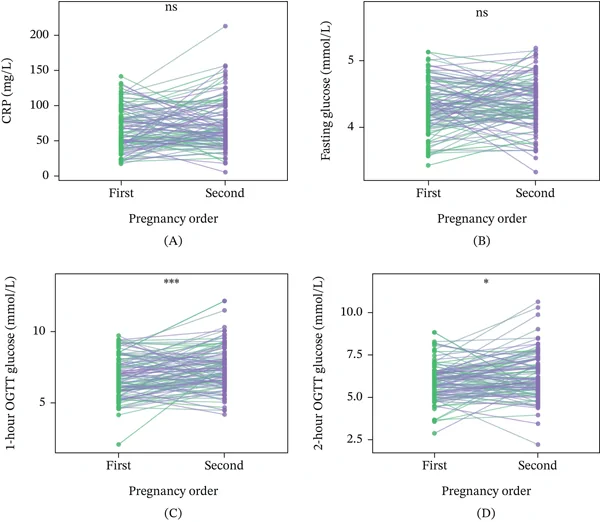
(A) Comparison of CRP between first and second pregnancies. CRP, C‐reactive protein *n* = 108. (B) Comparison of OGTT0h between first and second pregnancies. OGTT0h: fasting glucose. *n* = 108. (C) Comparison of OGTT1h between first and second pregnancies. OGTT1h:1‐h OGTT glucose. *n* = 108. (D) Comparison of OGTT2h between first and second pregnancies. OGTT2h: 2‐h OGTT glucose. *n* = 108. ∗: *p* value < 0.05, ∗∗∗: *p* value < 0.001. First: the first pregnancy. Second: the second pregnancy.

### 3.2. Result 2: Renal Function and Metabolic Parameter Shifts Across Pregnancies

The BUN/Scr was significantly higher during the second pregnancy compared with the first (median 9.75 vs. 9.89, *p* < 0.001, Figure 3A). Yet LDH is a marker of tissue turnover and metabolic stress. It did not show any significant variation between pregnancies (median 152.1 vs. 154.6 U/L, *p* = 0.192, Figure 3B). The levels of urea and other indicators of kidney function remained largely the same between the first and second pregnancies (median 2.7 vs. 2.64 mmol/L, *p* = 0.185; Figure 3C). In contrast, notably, maternal serum creatinine during pregnancy showed a clear and statistically significant decline in the second pregnancy (median 67.7 vs. 65.1 *μ*mol/L, *p* < 0.001, Figure 3D). Yet uric acid levels stayed the same (mean 268.79 vs. 279.38 *μ*mol/L, *p* = 0.119; Figure 3E).

**Figure 3:**
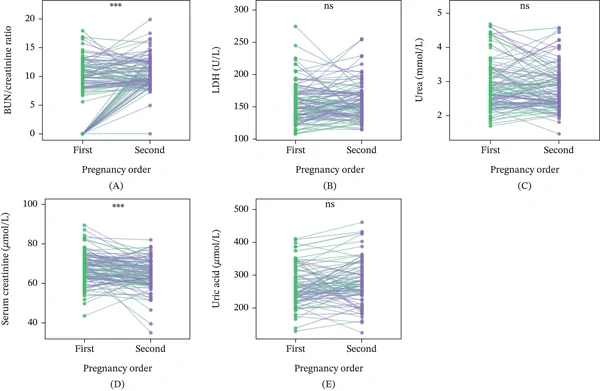
(A) Comparison of BUN/creatinine ratio between first and second pregnancies. BUN, blood urea nitrogen. *n* = 107. (B) Comparison of LDH between first and second pregnancies. LDH, lactate dehydrogenase. *n* = 105. (C) Comparison of urea between first and second pregnancies. *n* = 105. (D) Comparison of serum creatinine between first and second pregnancies. *n* = 107. (E) Comparison of uric acid between first and second pregnancies. *n* = 82. ∗∗∗: *p* value < 0.001. First: the first pregnancy. Second: the second pregnancy.

### 3.3. Result 3: Changes in Neonatal and Maternal Parameters

For neonatal parameters, the mean birth weight was lower in the second pregnancy than in the first. (median 3630 g vs. 3525 g, *p* = 0.034, Figure 4A). Notably, maternal weight was lower in the second pregnancy than in the first (median 74.75 vs. 73.00 kg, *p* = 0.008, Figure 4B). As expected, maternal age was higher among second pregnancies (median 28 vs. 32 years, *p* < 0.001, Figure 4C). Importantly, maternal BMI was lower in second pregnancies relative to first pregnancies (mean 28.11 vs. 27.72, *p* = 0.028, Figure 4D).

**Figure 4:**
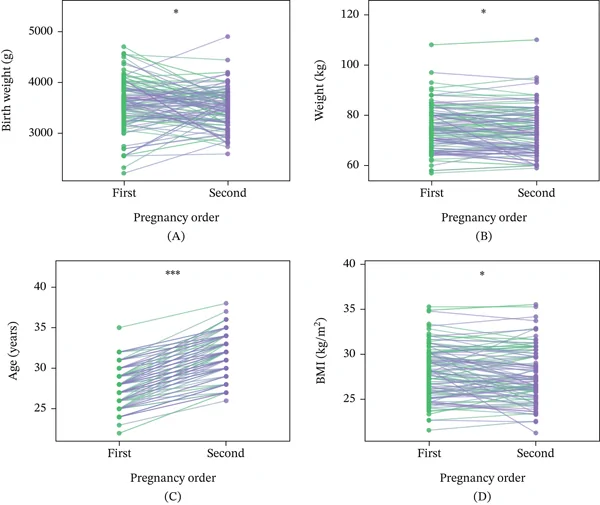
(A) Comparison of birth weight between first and second pregnancies. *n* = 108. (B) Comparison of weight between first and second pregnancies. *n* = 106. (C) Comparison of age between first and second pregnancies. Age: maternal age. *n* = 108. (D) Comparison of BMI between first and second pregnancies. BMI, body mass index. *n* = 106.∗: *p* value < 0.05, ∗∗∗: *p* value < 0.001. First: the first pregnancy. Second: the second pregnancy.

Considering changes in glucose metabolism, in the second pregnancy, both the 1‐ and 2‐h plasma glucose concentrations at 24–28 gestational weeks on the 75‐g OGTT were significantly higher than in the first pregnancy. This occurred alongside a decrease in maternal weight and BMI in the second pregnancy, indicating a drastic deterioration in postprandial glycemic control across pregnancies. Table S5 shows the proportions of patients achieving clinically meaningful thresholds across all assessed domains: inflammatory markers, glucose metabolism changes, renal function, metabolic parameters, and neonatal and maternal parameters.

## 4. Discussion

In this paired longitudinal analysis of women undergoing two cesarean deliveries, we identified several key changes within individuals between pregnancies: a significant increase in 2‐h plasma glucose after a 75‐g glucose load, an increase in the BUN/Scr, and a decrease in weight, BMI, and birth weight. Notably, postoperative CRP levels, fasting glucose values, and serum urea levels showed no statistically significant differences between the first and second cesarean deliveries. These results imply that, although systemic inflammation following cesarean delivery appears to be consistent between pregnancies, subtle alterations in glucose tolerance and renal function markers may occur over time.

The elevation in 2‐h plasma glucose after a 75‐g glucose load in the second pregnancy, in the absence of significant changes in fasting values, aligns with evidence that the 1‐ and 2‐h glucose loads are sensitive indicators of *β*‐cell secretory dysfunction and hepatic insulin resistance [11, 17]. Several cohorts have demonstrated that a higher 1‐ and 2‐h plasma glucose after a 75‐g glucose load value, even within normal limits, predicts postpartum dysglycemia and long‐term Type 2 diabetes risk [18, 19]. Mechanistically, the second pregnancy was associated with higher postload glucose values, suggesting possible subtle changes in postprandial glucose handling [20, 21]. As our group of women did not include those with GDM in their first pregnancy, this pattern shows the importance of checking for problems with blood sugar in the second pregnancy, especially if they are losing weight and their BMI is getting lower.

There were also noticeable changes in kidney function. The rise in BUN/Scr can indicate small changes in kidney blood flow or in how proteins are processed between pregnancies [22, 23]. Physiologically, pregnancy is associated with increased GFR and decreased serum creatinine [24, 25]; however, the rise in BUN/Scr levels could signal relative hypoperfusion, increased protein catabolism, or early tubular dysfunction. We have seen similar patterns linked to adverse pregnancy outcomes (including maternal end‐organ injury, fetal growth restriction, placental abruption, and fetal demise) in pre‐eclampsia [26], but none of our participants had severe high blood pressure (systolic blood pressure of ≥ 160 mmHg and/or diastolic blood pressure of ≥ 110 mmHg after 20 weeks of gestation, with or without proteinuria). Although the drop in creatinine is normal during pregnancy [27], it is not a specific marker of increased blood volume. Rather pregnancy‐related plasma volume expansion and increased GFR can lower the serum creatinine values. However, these mechanistic interpretations remain speculative. Clinically, further assessment of renal function—including estimated GFR, urine protein, blood pressure, hydration status, and albumin—would be necessary to better evaluate any potential renal changes. The results have suggested that renal parameters warrant closer monitoring in pregnancies following a first cesarean delivery.

From a clinical perspective, these findings have several implications. First, the rise in 2‐h plasma glucose after a 75‐g glucose load suggests that standard GDM screening may underestimate early dysglycemia in multiparous women without prior GDM. Incorporating 1‐ and 2‐h glucose levels as continuous risk markers, rather than a dichotomous threshold, could improve the prediction of adverse metabolic outcomes [28]. Second, monitoring the BUN/Scr across pregnancies may help identify women with subclinical renal stress [29, 30], who could benefit from closer surveillance or lifestyle interventions. Thirdly, the CRP levels were not statistically significant between the first and second cesarean deliveries. This suggests that the inflammatory responses after the operation are stable and probably not the reason for the observed differences in metabolism. This finding shows that internal physiological changes are more important than differences in surgery.

Strengths of this study include the intraindividual design, standardized sampling time points, and exclusion of obstetric confounders such as infection and preterm labor. However, several limitations should be acknowledged. First, selection bias may arise from the retrospective design and missing core data. Participants with missing core metabolic data were excluded, leading to a relatively high exclusion rate. This has two main implications: the final cohort had relatively low baseline risk, so the adverse metabolic effects of repeated pregnancies may be underestimated; and our findings cannot be generalized to all high‐risk women with comorbidities, infections or incomplete clinical records. Notably, even within this low‐risk cohort, marked alterations in glucose metabolism and renal function were observed. Women with metabolic risk factors are prone to renal and other complications and need close clinical monitoring. We investigated metabolic changes among women without comorbidities after two cesarean deliveries. Excluding high‐risk cases and patients with missing core data was necessary to minimize confounding, although this screening led to substantial sample loss and inevitable selection bias. Future larger multicenter cohort studies recruiting women across all gestational risk strata are needed to validate our findings and mitigate selection bias. Second, we lacked detailed data on diet and physical activity. No standardized grading system was available to quantify operative time, blood loss, and surgical complexity between primary and repeat cesarean sections; these unmeasured factors may have influenced our findings. We could not evaluate pancreatic *β*‐cell function due to unavailable insulin and C‐peptide measurements. We also did not assess additional inflammatory and hormonal mediators (e.g., IL‐6, adiponectin, placental hormones) that may account for the observed biochemical changes. Third, all participants underwent cesarean delivery, so our findings may lack generalizability to other delivery populations. Furthermore, the absence of postpartum metabolic assessment precluded evaluation of long‐term metabolic outcomes.

In summary, this study identified small but statistically significant within‐woman changes from the first to the second pregnancy in 2‐h plasma glucose during a 75‐g OGTT, BUN/Scr, serum creatinine, BMI, and maternal weight. These findings may support closer monitoring of renal parameters and glucose metabolism in a second pregnancy after cesarean delivery, particularly in women who experienced declines in maternal weight and BMI, with particular attention to glucose monitoring. Further longitudinal studies are needed to clarify the underlying mechanisms and to inform clinical care for women with multiple pregnancies; such studies should assess hormonal, inflammatory, vascular, and adipose tissue markers.

## 5. Conclusion

In this paired within‐subject study of women without overt GDM in the first pregnancy who underwent two consecutive cesarean deliveries, small but statistically significant within‐woman changes were observed from the first to the second pregnancy in 2‐h plasma glucose during a 75‐g OGTT, BUN/Scr, serum creatinine, BMI, and maternal weight. The increase in 2‐h plasma glucose suggests subtle changes in postprandial glucose handling, whereas the concurrent increase in the BUN/Scr and decrease in serum creatinine may indicate changes in renal adaptation, possibly reflecting altered renal perfusion or nitrogen balance. The paired design reduced interindividual variability and enabled assessment of within‐woman changes. These findings may support individualized risk assessment and longitudinal monitoring of glucose metabolism and renal function, particularly in women with a history of cesarean delivery and declining weight and BMI.

Future studies should assess insulin sensitivity and renal function, and further investigate inflammatory pathways. Changes in maternal weight, dietary intake, and lifestyle behaviors between pregnancies should also be evaluated. Such studies may help identify women who may benefit from closer monitoring in subsequent pregnancies.

## Author Contributions

Rui Chen: conceptualization, methodology, investigation, visualization, supervision, writing—original draft, writing—review and editing. Lin Xu: conceptualization and supervision. Chao Li: conceptualization and supervision. Yan Li: methodology, visualization, writing—original draft. Chunling Ma: investigation. Jie Chen: investigation. Qun Gao: investigation. Yu Zhang: visualization, writing—original draft. Yu Zhang and Yan Li have contributed to the work equally and should be regarded as co‐first authors.

## Funding

This study was supported by the National Natural Science Foundation of Qingdao Municipality (24‐4‐4‐zrjj‐119‐jch); the Taishan Scholar Project of Shandong Province (10.13039/501100010040; tsqn202306399); and the Natural Science Foundation of Shandong Province (10.13039/501100007129; ZR2024QH209).

## Disclosure

All authors have read and approved the final version of the manuscript. Rui Chen has full access to all of the data in this study and takes complete responsibility for the integrity of the data and the accuracy of the data analysis.

## Ethics Statement

The study was approved by the Ethics Committee of Qingdao University Affiliated Hospital (QYFY‐WZLL‐29236) and was conducted in accordance with the Declaration of Helsinki and applicable institutional requirements.

## Conflicts of Interest

The authors declare no conflicts of interest.

## Supporting information


**Supporting Information** Additional supporting information can be found online in the Supporting Information section. Table S1: Wilcoxon signed‐rank test of nonnormally distributed variables. Table S2: Paired‐sample *t*‐tests of normally distributed variables. Table S3: Paired *t*‐test for OGTT values (women without GDM in both pregnancies). Table S4: Linear mixed model adjusted for baseline age, interpregnancy age increment, BMI change, and gestational age on the day prior to laboratory examination. Table S5: Proportion of patients reaching clinically meaningful thresholds.

## Data Availability

The data that support the findings of this study are available from the corresponding authors upon reasonable request.

## References

[1] Tan E. K. and Tan E. L. , Alterations in Physiology and Anatomy During Pregnancy, Best Practice & Research Clinical Obstetrics & Gynaecology. (2013) 27, no. 6, 791–802, 10.1016/j.bpobgyn.2013.08.001. 24012425

[2] Wang H. , Li N. , Chivese T. , Werfalli M. , Sun H. , Yuen L. , Hoegfeldt C. A. , Elise Powe C. , Immanuel J. , Karuranga S. , Divakar H. , Levitt N. , Li C. , Simmons D. , Yang X. , and IDF Diabetes Atlas Committee Hyperglycaemia in Pregnancy Special Interest Group , IDF Diabetes Atlas: Estimation of Global and Regional Gestational Diabetes Mellitus Prevalence for 2021 by International Association of Diabetes in Pregnancy Study Group′s Criteria, Diabetes Research and Clinical Practice. (2022) 183, 109050, 10.1016/j.diabres.2021.109050, 34883186. 34883186

[3] Johns E. C. , Denison F. C. , Norman J. E. , and Reynolds R. M. , Gestational Diabetes Mellitus: Mechanisms, Treatment, and Complications, Trends in Endocrinology & Metabolism. (2018) 29, no. 11, 743–754, 10.1016/j.tem.2018.09.004. 30297319

[4] Vounzoulaki E. , Khunti K. , Abner S. C. , Tan B. K. , Davies M. J. , and Gillies C. L. , Progression to Type 2 Diabetes in Women With a Known History of Gestational Diabetes: Systematic Review and Meta-Analysis, British Medical Journal. (2020) 369, m1361, 10.1136/bmj.m1361, 32404325. PMC7218708 32404325

[5] Chen L. , Cesarean Section Indication and Perinatal Outcomes of Infants With Macrosomia in Primiparous Women: A Retrospective Cohort Study, Clinical and Experimental Obstetrics & Gynecology. (2025) 52, no. 12, 42782, 10.31083/ceog42782.

[6] Boerma T. , Ronsmans C. , Melesse D. Y. , Barros A. J. D. , Barros F. C. , Juan L. , Moller A. B. , Say L. , Hosseinpoor A. R. , Yi M. , de Lyra Rabello D. , and Temmerman M. , Global Epidemiology of Use of and Disparities in Caesarean Sections, Lancet. (2018) 392, no. 10155, 1341–1348, 10.1016/s0140-6736(18)31928-7. 30322584

[7] Betran A. P. , Ye J. , Moller A. B. , Souza J. P. , and Zhang J. , Trends and Projections of Caesarean Section Rates: Global and Regional Estimates, BMJ Global Health. (2021) 6, no. 6, e005671, 10.1136/bmjgh-2021-005671, 34130991. PMC8208001 34130991

[8] Nittiwatthanawit P. and Suriyachai P. , Comparison of a Modified Enhanced Recovery After Surgery Protocol With the Standard Protocol in Emergency Cesarean Deliveries: A Randomized Controlled Trial, Clinical and Experimental Obstetrics & Gynecology. (2025) 52, no. 10, 42810, 10.31083/ceog42810.

[9] Tita A. T. , Szychowski J. M. , Boggess K. , Saade G. , Longo S. , Clark E. , Esplin S. , Cleary K. , Wapner R. , Letson K. , Owens M. , Abramovici A. , Ambalavanan N. , Cutter G. , Andrews W. , and C/SOAP Trial Consortium , Adjunctive Azithromycin Prophylaxis for Cesarean Delivery, New England Journal of Medicine. (2016) 375, no. 13, 1231–1241, 10.1056/NEJMoa1602044, 27682034. PMC5131636 27682034

[10] Tian Q. , Zhang Y. , Huang X. , Wang W. , Chen T. , and Chen R. , Molecular Mechanisms of Arcus Tendineus Fascia Pelvis Degeneration in Pelvic Floor Dysfunction: A Narrative Review, Clinical and Experimental Obstetrics & Gynecology. (2026) 53, no. 5, 47579, 10.31083/ceog47579.

[11] Miyakoshi K. , Tanaka M. , Saisho Y. , Shimada A. , Minegishi K. , Kim S. H. , Asai S. , Itoh H. , and Yoshimura Y. , Pancreatic *β*‐cell Function and Fetal Growth in Gestational Impaired Glucose Tolerance, Acta Obstetricia et Gynecologica Scandinavica. (2010) 89, no. 6, 769–775, 10.3109/00016349.2010.487091. 20504080

[12] Huhn E. A. , Kotzaeridi G. , Fischer T. , Todesco Bernasconi M. , Richter A. S. , Kunze M. , Dölzlmüller E. , Jaksch-Bogensperger H. , Weidinger L. , Eppel D. , Ochsenbein-Koelble N. , Bäz E. , Winzeler B. , Tura A. , Stach H. , Schäfer G. , van Breda S. V. , Vokálová L. , Hoesli I. , and Göbl C. S. , The Utility of Early Gestational OGTT and Biomarkers for the Development of Gestational Diabetes Mellitus: An International Prospective Multicentre Cohort Study, Diabetologia. (2025) 68, no. 11, 2511–2522, 10.1007/s00125-025-06517-0, 40817933. PMC12534333 40817933

[13] Retnakaran R. , Zinman B. , Connelly P. W. , Sermer M. , and Hanley A. J. , Impaired Glucose Tolerance of Pregnancy Is a Heterogeneous Metabolic Disorder as Defined by the Glycemic Response to the Oral Glucose Tolerance Test, Diabetes Care. (2006) 29, no. 1, 57–62, 10.2337/diacare.29.1.57, 16373896. 16373896

[14] Cheung K. L. and Lafayette R. A. , Renal Physiology of Pregnancy, Advances in Chronic Kidney Disease. (2013) 20, no. 3, 209–214, 10.1053/j.ackd.2013.01.012, 23928384. PMC4089195 23928384

[15] Kavak E. C. , Akcabay C. , Demircan M. , Batmaz I. , Sanli C. , Senocak A. , Haliscelik M. A. , Onat M. , Tepe B. , and Kavak S. B. , The Role of Mid-Trimester BUN and Creatinine Assessment in Predicting Preeclampsia: Retrospective Case-Control Study, Medicina. (2025) 61, no. 4, 10.3390/medicina61040746, 40283037. PMC12028929 40283037

[16] Gong Z. , Yue H. , Li Z. , Bai S. , Cheng Z. , He J. , Wang H. , Li G. , and Sang N. , Association Between Maternal Exposure to Air Pollution and Gestational Diabetes Mellitus in Taiyuan, North China, Science of the Total Environment. (2023) 875, 162515, 10.1016/j.scitotenv.2023.162515, 36868286. 36868286

[17] Retnakaran R. , Qi Y. , Sermer M. , Connelly P. W. , Zinman B. , and Hanley A. J. , Isolated Hyperglycemia at 1 Hour on Oral Glucose Tolerance Test in Pregnancy Resembles Gestational Diabetes Mellitus in Predicting Postpartum Metabolic Dysfunction, Diabetes Care. (2008) 31, no. 7, 1275–1281, 10.2337/dc08-0126, 18356402. PMC2453676 18356402

[18] Retnakaran R. , Qi Y. , Sermer M. , Connelly P. W. , Hanley A. J. , and Zinman B. , Glucose Intolerance in Pregnancy and Future Risk of Pre-Diabetes or Diabetes, Diabetes Care. (2008) 31, no. 10, 2026–2031, 10.2337/dc08-0972, 18628572. PMC2551649 18628572

[19] Carr D. B. , Newton K. M. , Utzschneider K. M. , Tong J. , Gerchman F. , Kahn S. E. , and Heckbert S. R. , Modestly Elevated Glucose Levels During Pregnancy Are Associated With a Higher Risk of Future Diabetes Among Women Without Gestational Diabetes Mellitus, Diabetes Care. (2008) 31, no. 5, 1037–1039, 10.2337/dc07-1957, 18223032. 18223032

[20] Jensen C. C. , Cnop M. , Hull R. L. , Fujimoto W. Y. , Kahn S. E. , and the American Diabetes Association GENNID Study Group , Beta-Cell Function Is a Major Contributor to Oral Glucose Tolerance in High-Risk Relatives of Four Ethnic Groups in the U.S, Diabetes. (2002) 51, no. 7, 2170–2178, 10.2337/diabetes.51.7.2170, 12086947. 12086947

[21] Ergin T. , Lembet A. , Duran H. , Kuscu E. , Bagis T. , Saygili E. , and Batioglu S. , Does Insulin Secretion in Patients With One Abnormal Glucose Tolerance Test Value Mimic Gestational Diabetes Mellitus?, American Journal of Obstetrics and Gynecology. (2002) 186, no. 2, 204–209, 10.1067/mob.2002.119634, 11854636. 11854636

[22] Han L. , Yang Z. , Li K. , Zou J. , Li H. , Han J. , Zhou L. , Liu X. , Zhang X. , Zheng Y. , Yu L. , and Li L. , Antepartum or Immediate Postpartum Renal Biopsies in Preeclampsia/Eclampsia of Pregnancy: New Morphologic and Clinical Findings, International Journal of Clinical and Experimental Pathology. (2014) 7, no. 8, 5129–5143, 25197387. PMC4152077 25197387

[23] Brisco M. A. , Coca S. G. , Chen J. , Owens A. T. , McCauley B. D. , Kimmel S. E. , and Testani J. M. , Blood Urea Nitrogen/Creatinine Ratio Identifies a High-Risk but Potentially Reversible Form of Renal Dysfunction in Patients With Decompensated Heart Failure, Circulation: Heart Failure. (2013) 6, no. 2, 233–239, 10.1161/circheartfailure.112.968230, 23325460. PMC4067251 23325460

[24] Abram S. R. , Alexander B. T. , Bennett W. A. , and Granger J. P. , Role of Neuronal Nitric Oxide Synthase in Mediating Renal Hemodynamic Changes During Pregnancy, American Journal of Physiology-Regulatory, Integrative and Comparative Physiology. (2001) 281, no. 5, R1390–R1393, 10.1152/ajpregu.2001.281.5.R1390, 11641107. 11641107

[25] Jeyabalan A. and Conrad K. P. , Renal Function During Normal Pregnancy and Preeclampsia, Frontiers in Bioscience. (2007) 12, no. 1, 2425–2437, 10.2741/2244. 17127252

[26] American College of Obstetricians and Gynecologists , ACOG Practice Bulletin No. 203: Chronic Hypertension in Pregnancy, Obstetrics & Gynecology. (2019) 133, no. 1, e26–e50, 10.1097/aog.0000000000003018. 30575676

[27] Maynard S. E. and Thadhani R. , Pregnancy and the Kidney, Journal of the American Society of Nephrology. (2009) 20, no. 1, 14–22, 10.1681/asn.2008050493. 19118150

[28] Cheng X. , Yang N. , Li Y. , Sun Q. , Qiu L. , Xu L. , Ping F. , Li W. , and Zhang H. , The Shape of the Glucose Response Curve During an Oral Glucose Tolerance Test Heralds *β*-Cell Function in a Large Chinese Population, BMC Endocrine Disorders. (2019) 19, no. 1, 10.1186/s12902-019-0446-4, 31690291. PMC6833144 31690291

[29] Inaguma D. , Koide S. , Ito E. , Takahashi K. , Hayashi H. , Hasegawa M. , and Yuzawa Y. , Ratio of Blood Urea Nitrogen to Serum Creatinine at Initiation of Dialysis Is Associated With Mortality: A Multicenter Prospective Cohort Study, Clinical and Experimental Nephrology. (2018) 22, no. 2, 353–364, 10.1007/s10157-017-1458-x, 28766029. 28766029

[30] Uchino S. , Bellomo R. , and Goldsmith D. , The Meaning of the Blood Urea Nitrogen/Creatinine Ratio in Acute Kidney Injury, Clinical Kidney Journal. (2012) 5, no. 2, 187–191, 10.1093/ckj/sfs013, 29497527. PMC5783213 29497527

