# Supplementary material for: Glucose Metabolism, Renal Indices, and C‐Reactive Protein Across Two Cesarean Deliveries in the Same Women: A Retrospective Paired Cohort Study
Source: J Diabetes Res. 2026 Nov 6;2026:2449846. doi: 10.1155/jdr/2449846 (PMC13741291; doi:10.1155/jdr/2449846)
Supplement: Supplementary file 1 [file JDR-2026-2449846-s001.docx]

| Supplementary Table S1. Wilcoxon Signed-Rank Test of Non-Normally Distributed Variables. | | | | |
| --- | --- | --- | --- | --- |
| Variables | Median (IQR) | r | Mean difference(95% CI lower,95% CI upper) | P |
| CRP1 (mg/L) | 62.58 (46.73, 92.66) | 0.147 | -6.141(-13.099, 0.817) | 0.126 |
| CRP2 (mg/L) | 65.44 (49.68, 97.29) |  |  |  |
| OGTT2h1 (mmol/L) | 5.99 (5.17, 6.45) | 0.246 | -0.320(-0.574, -0.066) | 0.011 |
| OGTT2h2 (mmol/L) | 6.03 (5.41, 7.22) |  |  |  |
| weight1 (kg) | 74.75(68.75, 80.25) | 0.258 | 1.346(0.450, 2.243) | 0.008 |
| weight2 (kg) | 73.00 (66.00, 80.00) |  |  |  |
| Urea1(mmol/L) | 2.70 (2.38, 3.32) | 0.129 | 0.097(-0.024, 0.218) | 0.185 |
| Urea2(mmol/L) | 2.64 (2.34, 3.14) |  |  |  |
| BUN_Scr1 | 9.75 (5.61, 11.73) | 0.389 | -2.434(-3.399, -1.470) | ＜0.001 |
| BUN_Scr2 | 9.89 (8.77, 12.42) |  |  |  |
| Creatinine1(μmol/L) | 67.70 (63.10, 72.70) | 0.372 | 3.125(1.516, 4.734) | ＜0.001 |
| Creatinine2（μmol/L) | 65.10 (60.00, 69.4) |  |  |  |
| LDH1(U/L) | 152.10 (133.45, 172.30) | 0.127 | -2.789(-8.627, 3.050) | 0.192 |
| LDH2(U/L) | 154.60 (138.50, 172.00) |  |  |  |
| Birth_weight1 (g) | 3630(3322.5, 3915) | 0.204 | 93.843(-0.503, 188.188) | 0.034 |
| Birth_weight2 (g) | 3525(3222.5, 3760) |  |  |  |
| age1 (Years) | 28(26, 29) | 0.874 | -3.8780(-4.170, -3.589) | ＜0.001 |
| age2 (Years) | 32(30, 34) |  |  |  |
| interquartile range (IQR): Q1, 25th percentile, Q3, 75th percentile; Variables1: variables of the first pregnancy; Variables2: variables of the second pregnancy; BUN_Scr: Blood Urea Nitrogen to Serum Creatinine Ratio. CRP indicates stimulated C-reactive protein measured 24-48 hours post-cesarean section. OGTT2h represent glucose levels at 2 hours respectively during a 75-g OGTT performed at 24-28 weeks of gestation. Urea levels reflect nitrogen metabolism, while BUN_Scr represents the ratio of blood urea nitrogen to serum creatinine, an indicator of renal filtration dynamics. LDH (lactate dehydrogenase) serves as a general marker of tissue turnover. A P value < 0.05 was considered statistically significant. CI: Confidence Interval; lower 95% CI : lower bound of the 95% confidence interval：upper 95% CI: upper bound of the 95% confidence interval. r: effect size. Mean differences = Mean(variables1）-Mean(variables2) | | | | |

| Supplementary Table S2. Paired-sample t-tests of Normally Distributed Variables. | | | | | |
| --- | --- | --- | --- | --- | --- |
| Variables | Mean±SD | t | Cohens’d | Mean difference(95% CI lower,95% CI upper) | P |
| OGTT0h1(mmol/L) | 4.32±0.38 | -1.072 | 0.103 | -0.042(-0.121, 0.036) | 0.286 |
| OGTT0h2(mmol/L) | 4.36±0.37 |  |  |  |  |
| OGTT1h1(mmol/L) | 6.81±1.43 | -4.175 | 0.402 | -0.623(-0.918, -0.327) | ＜0.001 |
| OGTT1h2(mmol/L) | 7.43±1.54 |  |  |  |  |
| Uric acid1(μmol/L) | 268.79±60.74 | -1.575 | 0.174 | -10.596(-23.984, 2.792) | 0.119 |
| Uric acid2(μmol/L) | 279.38±69.40 |  |  |  |  |
| BMI1(kg/m²) | 28.11±2.87 | 2.224 | 0.216 | 0.396(0.043, 0.750) | 0.028 |
| BMI2(kg/m²) | 27.72±2.94 |  |  |  |  |
| SD: Standard Deviation Variables1: variables of the first pregnancy; Variables2: variables of the second pregnancy. Uric acid levels reflect nitrogen metabolism. OGTT0h and OGTT1h represent glucose levels at 0 and 1 hours respectively during a 75-g OGTT performed at 24-28 weeks of gestation. A P value < 0.05 was considered statistically significant. CI: Confidence Interval; lower 95% CI : lower bound of the 95% confidence interval：upper 95% CI: upper bound of the 95% confidence interval. Mean differences = Mean(variables1)-Mean(variables2) | | | | | |

| Supplementary Table S3. Paired t-test for OGTT values (women without GDM in both pregnancies) | | | | | |
| --- | --- | --- | --- | --- | --- |
| Variables | Mean±SD | t | Cohens’d | Mean difference(95% CI lower,95% CI upper) | P |
| OGTT0h1(mmol/L) | 4.33±0.37 | 0.354 | 0.036 | 0.013(-0.062, 0.089) | 0.724 |
| OGTT0h2(mmol/L) | 4.31±0.35 |  |  |  |  |
| OGTT1h1(mmol/L) | 6.64±1.40 | -3.15 | 0.320 | -0.489(-0.797, -0.181) | 0.002 |
| OGTT1h2(mmol/L) | 7.13±1.25 |  |  |  |  |
| OGTT2h1(mmol/L) | 5.80±1.04 | -2.12 | 0.216 | -0.247(-0.478, -0.016) | 0.036 |
| OGTT2h2(mmol/L) | 6.04±1.11 |  |  |  |  |
| SD: Standard Deviation Variables1: variables of the first pregnancy; Variables2: variables of the second pregnancy. OGTT0h and OGTT1h represent glucose levels at 0 and 1 hours respectively during a 75-g OGTT performed at 24-28 weeks of gestation. OGTT2h represent glucose levels at 2 hours respectively during a 75-g OGTT performed at 24-28 weeks of gestation. GDM:Gestational Diabetes Mellitus. A P value < 0.05 was considered statistically significant. | | | | | |

| Supplementary Table S4. Linear mixed model adjusted for baseline age, inter-pregnancy age increment, BMI change, and gestational age on the day prior to laboratory examination | | |
| --- | --- | --- |
| Variables | F | P |
| CRP (mg/L) | 3.631 | 0.059 |
| OGTT0h(mmol/L) | 0.049 | 0.825 |
| OGTT1h(mmol/L) | 1.498 | 0.223 |
| OGTT2h(mmol/L) | 4.632 | 0.033 |
| Urea(mmol/L) | 0.147 | 0.702 |
| Creatinine (μmol/L) | 0.018 | 0.894 |
| LDH (U/L) | 0.229 | 0.633 |
| Birth_weight (g) | 0.533 | 0.467 |
| weight (kg) | 0.148 | 0.701 |
| Uric acid (μmol/L) | 1.439 | 0.234 |
| Variables1: variables of the first pregnancy; Variables2: variables of the second pregnancy; BUN_Scr: Blood Urea Nitrogen to Serum Creatinine Ratio. CRP indicates stimulated C-reactive protein measured 24-48 hours post-cesarean section. OGTT0h and OGTT1h represent glucose levels at 0 and 1 hours respectively during a 75-g OGTT performed at 24-28 weeks of gestation. OGTT2h represent glucose levels at 2 hours respectively during a 75-g OGTT performed at 24-28 weeks of gestation. Urea levels reflect nitrogen metabolism, while BUN_Scr represents the ratio of blood urea nitrogen to serum creatinine, an indicator of renal filtration dynamics. LDH (lactate dehydrogenase) serves as a general marker of tissue turnover. A P value < 0.05 was considered statistically significant. We identified 26 extreme outliers with a BUN-Scr ratio < 0.1 among the measurements. To evaluate the robustness of our results, these outliers were excluded, and the mixed-linear model was refitted (the BUN-Scr ratio of the remaining participants ranged from 5.61 to 17.94). This re-analysis confirmed a significant, robust difference in 2-hour post-load plasma glucose across pregnancy groups. | | |

| Supplementary Table S5.Proportion of patients reaching clinically meaningful thresholds | |
| --- | --- |
| Variables | Proportion |
| CRP1 (mg/L) | 100% |
| CRP2 (mg/L) | 99.07% |
| OGTT0h1(mmol/L) | 0% |
| OGTT0h2(mmol/L) | 1.85% |
| OGTT1h1(mmol/L) | 0% |
| OGTT1h2(mmol/L) | 4.62% |
| OGTT2h1(mmol/L) | 0% |
| OGTT2h2(mmol/L) | 3.70% |
| Urea1(mmol/L) | 0% |
| Urea2(mmol/L) | 0% |
| BUN_Scr1 | 0% |
| BUN_Scr2 | 0% |
| Creatinine1(μmol/L) | 6.54% |
| Creatinine2（μmol/L) | 0.93% |
| LDH1(U/L) | 0.95% |
| LDH2(U/L) | 1.90% |
| Uric acid1(μmol/L) | 6.09% |
| Uric acid2(μmol/L) | 13.41% |
| BMI1(kg/m²) | 94.34% |
| BMI2(kg/m²) | 90.57% |
| SD: Standard Deviation Variables1: variables of the first pregnancy; Variables2: variables of the second pregnancy. OGTT0h and OGTT1h represent glucose levels at 0 and 1 hours respectively during a 75-g OGTT performed at 24-28 weeks of gestation. GDM:Gestational Diabetes Mellitus. A P value < 0.05 was considered statistically significant. CRP(C-reactive protein) (mg/L): Levels above 10 mg/L suggest a risk of inflammation and infection (clinically significant). CRP >10 mg/L suggests infection risk, but postpartum infection evaluation must consider maternal temperature and clinical findings. OGTT 0h(Fasting blood glucose) (mmol/L): Values above 5.1 mmol/L indicate abnormal glucose metabolism with clinical significance. OGTT 1h(1-hour postprandial blood glucose) (mmol/L): Values above 10.0 mmol/L indicate impaired glucose tolerance with clinical significance. OGTT 2h (2-hour postprandial blood glucose) (mmol/L): Values above 8.5 mmol/L indicate impaired glucose tolerance with clinical significance. Urea1(Blood urea) (mmol/L): Values above 7.5 mmol/L indicate abnormal renal metabolism with clinical significance. BUN_Scr: Ratios above 25 indicate renal function imbalance and abnormal renal perfusion with clinical significance. Creatinine: Values above 81 μmol/L indicate renal impairment with clinical significance.  Uric acid1, Uric acid (μmol/L): Values above 357 μmol/L indicate hyperuricemia with clinical significance. LDH(U/L): Persistent levels above 250 U/L suggest tissue damage and visceral abnormalities with clinical significance. BMI (Body Mass Index) (kg/m²): BMI ≥24 indicates overweight. BMI is calculated based on the maternal height and weight measured on the day before cesarean section. | |
